# The Pattern of Superficial Lymphadenopathy on Fine Needle Aspiration Cytology in Clinical Practice in Islamabad

**DOI:** 10.7759/cureus.17075

**Published:** 2021-08-10

**Authors:** Nadeem Islam Sheikh, Mehreen Babar, Ambreen Zahoor, Zaidan Idrees, Sajid Naseem, Saba Fatima

**Affiliations:** 1 Internal Medicine, Social Security Hospital, Islamabad, PAK; 2 Ear, Nose, and Throat, Hazrat Bari Imam Sarkar (HBS) Medical College, Islamabad, PAK; 3 Medicine, Hazrat Bari Imam Sarkar (HBS) Medical College, Islamabad, PAK; 4 Psychiatry, Hazrat Bari Imam Sarkar (HBS) Medical College, Islamabad, PAK; 5 Medical Affairs and Clinical Research, Hilton Pharma Pvt. Ltd., Karachi, PAK

**Keywords:** lymphadenopathy, fine needle aspiration cytology (fnac), lymph node (ln), tuberculosis (tb), neoplastic

## Abstract

Background: In adults, lymph nodes are not normally palpable. A number of patients with asymptomatic lymphadenopathy never visit physicians for the condition, and thus, this important sign is often missed by the medical practitioner if it is not the presenting complaint. The incidence of lymphadenopathy is suggested to be increasing. While lymphadenopathy is benign and self-limiting in most patients, the underlying disease may range from treatable infectious etiology to malignant neoplasms. In most cases clinical examination and history guide towards the cause of lymphadenopathy. In recent years, fine needle aspiration cytology (FNAC) has become an easy clinical tool (with or without the assistance of CT, MRI, and ultrasound) for the diagnosis of the underlying cause of lymphadenopathy.

Aims and objectives: To find out the cytomorphological pattern in superficial lymphadenopathy with the help of FNAC.

Materials and methods: This descriptive cross-sectional study was conducted at HBS General Hospital, Islamabad from January 2017 to June 2019. Patients presenting with superficial lymphadenopathy were included in the study. FNAC was performed by the pathologist, histopathological reports were examined and analyzed using Statistical Package for the Social Sciences (SPSS) version 22 (IBM Corp., Armonk, NY).

Results: Six hundred and thirty-two patients underwent FNAC. Tuberculous lymphadenitis was the most common diagnosis (56.1%) followed by reactive hyperplasia (28.29%). The sample showed metastatic malignancy 3.36% and lymphoma 2.05%. Cervical lymphadenopathy was the most common site for TB (49.36%). Metastatic cancer observed in cervical lymph nodes was 3.16% and lymphoma was 1.74%.

Conclusion: FNAC is recognized as a simple and safe diagnostic technique that can diagnose cases of superficial and deep lymphadenopathy easily. The most common cause of superficial lymphadenopathy in our study was tuberculosis with cervical lymph nodes.

## Introduction

Lymphadenopathy, considered as an abnormality within the size or nature of lymph nodes, is produced by the invasion or dispersion of either inflammatory cells or neoplastic cells into the node. It results from an enormous array of disease processes [1]. Lymphadenopathy refers to enlarged lymph nodes and it is a common finding in clinical practice either as acute or chronic. Palpable lymph nodes greater than 5 mm are considered abnormal. The annual incidence of unexplained lymphadenopathy is 0.6%. Only 1.1% of cases are related to malignancy, the percentage of which increases with age [1]. About one-half of otherwise healthy children have palpable lymph nodes at any one time [2]. Lymphadenopathy in children is mostly benign or infectious in etiology. In adults and children, lymphadenopathy lasting less than two weeks or greater than 12 months without change in size is unlikely to be neoplastic in origin [3,4]. Exceptions include low-grade Hodgkin lymphoma and non-Hodgkin lymphoma both of which are associated with systemic symptoms [5].

An excision biopsy of the lymph node is the ideal investigation for diagnosis, but it requires local or generalized anesthesia. Fine needle aspiration cytology (FNAC) offers an alternative for diagnosis with little trauma and cost [6]. First FNAC was done in 1904 by two marine officers - Captain E.D.W. Greig and Lieutenant A.C.H. Grey. The first tumor diagnosis by FNAC was done in 1914 by English physician Gordon R in 1921. De May summarized the benefits of FNAC with the acronym SAFE (simple, accurate, fast, and economical). It can easily differentiate between malignant and nonmalignant lesions [5]. The diagnostic yield of FNAC can be improved if accompanied by radiological guidance like ultra-sonography and computed tomography scan [6].

In review studies of patients with lymphadenopathy, 17.5% were found to be malignant including 11.4% of lymphoproliferation and 6.1% of metastasis; 31% of cases were of reactive etiology and 26% had other non-malignant diseases [7]. A study conducted in Pakistan on 498 patients with cervical lymphadenopathy showed 8% had Hodgkin lymphoma (stages 2 and 3) [8]. The current study focused on cytomorphological patterns assessed by FNAC in cases of superficial lymphadenopathy presenting to a tertiary care hospital in Islamabad.

The rationale of the study

FNAC is recognized as a simple and safe diagnostic technique that can diagnose cases of superficial and deep lymphadenopathy access to tissue excision. This test may be recommended in the future as the first line of investigation. Results of the study also provide useful data which will help healthcare professionals in the future and improves the quality of life of the local community.

## Materials and methods

A descriptive cross-sectional study was conducted at HBS General Hospital, Islamabad Pakistan from January 2017 to June 2019. The study was approved by the ethical review committee of the hospital.

Participants

A total of 632 male and female patients were enrolled in the study between the ages of 15 to 70 years. Patients were selected and informed consent was taken from each participant. In the current study, all clinically diagnosed cases of superficial lymphadenopathy as per study protocol were included. Patients refusing to give consent were excluded from the study. Patients who are previously diagnosed with lymphadenopathy were also excluded from the study because lymph nodes will be difficult to aspirate. Patients on medication for lymphadenopathy and chemotherapy were also excluded from the study.

Data were gathered by examining lymph node biopsies. All clinically diagnosed cases of superficial lymphadenopathy who underwent FNAC as per study protocol were included. All patients had CP, erythrocyte sedimentation rate (ESR), C-reactive protein (CRP), Liver function tests (LFTs), hepatitis B surface antigen (HBS Ag), hepatitis C virus (HCV), and human immunodeficiency virus (HIV) tests done. X-ray chest and ultrasound abdomen were also conducted. The procedure was explained to every patient and written consent prior to the procedure were taken.

Statistics

The sample size for the study is calculated to be n = 632 patients at a 95% confidence level with a margin of error of 8%. Data were analyzed using SPSS version 21 (IMB, Inc., Armonk, USA). For quantitative data, mean and standard deviation were reported and for qualitative data frequencies and percentages were presented. Statistical significance was kept at α = 0.05.

## Results

Results of 632 lymph node biopsies were examined. In this study sample, 52.21% (330/632) of males and 47.7% (302/632) females had superficial lymphadenopathy. The minimum age of presentation was 15 years while the maximum was 70 years. Maximum patients 31.8% (201/632) presented between 21 and 30 years.

Cervical lymphadenopathy was the most common presentation comprising 85.4% (n=540) followed by axillary lymphadenopathy of 5.53% (n=35). Generalized lymphadenopathy was seen in 5.06% (n=32) and inguinal lymphadenopathy in 3.92% of patients (Tables 1-2). Tuberculous lymphadenitis was the most common isolated pathology seen in 56.0% (354/632) of males and 184 females. Tuberculosis was seen in 49.36% of cervical, 3.63% in axillary, 1.74% in generalized, and 1.26% in inguinal lymph nodes; 21.51% of patients with tuberculous lymphadenitis were between 21 and 30 years of age (Tables 1-3). Reactive hyperplasia of lymph nodes was seen in 242 (38.29%) cases with 145 males and 97 females (Table 1) with peak ages between 15 and 30 years. The cervical group of lymph nodes was the most common site for reactive hyperplasia seen in 196 (31%) cases. Also, 2.2% in inguinal lymph nodes, 1.9% in axillary, and 3.16% in generalized lymphadenopathy had reactive hyperplasia. Relevant clinical examination and laboratory tests were done to find the cause of reactive hyperplasia (Table 3).

**Table 1:** Cytological diagnosis on fine-needle aspiration cytology of the study sample

Histological diagnosis	Number	Percentage (%)
Tuberculous lymphadenitis	354	56.01
Reactive hyperplasia	242	38.29
Hodgkin lymphoma	10	1.58
Non-Hodgkin lymphoma	3	0.47
Metastatic malignancy	23	3.63

**Table 2:** Sites of lymph nodes in the study population HD: Hodgkin disease, NHD: non-Hodgkin disease.

Site	Reactive hyperplasia	Tuberculous lymphadenitis	Metastatic cancer	Lymphoma
Cervical	196 (31%)	312 (49.36%)	20 (3.16%)	HD: 9 (1.42%) NHD: 3 (0.47%)
Axillary	12 (1.9%)	23 (3.63%)	­­­­­­ -	-
Inguinal	14 (2.2%)	8 (1.26%)	2 (0.31)	HD: 1 (0.15%)
Generalized	20 (3.16%)	11 (1.74%)	1 (0.15%)	^-^

**Table 3:** Distribution of lymphadenopathy with age and gender

Age in years	Reactive hyperplasia (n 242)		TB lymphadenitis (n 354)		Lymphoma (n 13)		Metastatic cancer (n23)	
	M	F	M	F	M	F	M	F
15-20	58	40	69	56	2 (HD)	1 (HD)	-	-
21-30	36	24	57	79	3 (HD)	2 (HD)	-	-
31-40	30	16	27	36	1 (HD); 1(HD)	1 (NHD); 2 (NHD)	-	-
41-50	18	13	11	8	-	-	7	8
51-60	3	4	5	3	-	-	5	3
61-70	-	-	1	2	-	-	-	-

Hodgkin lymphoma was seen in 1.58% and non-Hodgkin lymphoma was seen in 0.47% of enrolled patients (Table 1). Hodgkin disease was more common between 21 and 30 years and non-Hodgkin was between 31 and 40 years. The frequency of prevalence of Hodgkin disease in cervical lymph nodes was 1.42% and non-Hodgkin disease was 0.47% (Tables 2-4). Inguinal lymphadenopathy had a prevalence of 0.15% Hodgkin disease. The maximum age of presentation with lymphoma was between 41 and 50 years with female predominance and cervical lymph node was the most common site (Tables 2-3); 3.63% of study participants had metastatic malignancy with cervical lymph nodes involved in 3.16% (Tables 1-2).

**Table 4:** Percentage distribution of isolated lymph nodes on fine-needle aspiration cytology

Site of lymph node	Reactive hyperplasia	TB lymphadenitis	Metastatic cancer	Lymphoma	
				HD	NHD
Cervical	31	49.36	3.16	1.42	0.47
Axillary	1.8	3.63	-	-	-
Inguinal	2.21	1.26	0.31	0.15	-
Generalized	3.16	1.74	0.158	-	-

## Discussion

The present study demonstrated sensitivity and overall specificity of 100% for diagnosing benign and malignant lesions in lymphadenopathy by FNAC. FNAC is a part of the initial diagnosis and management of patients presenting with lymphadenopathy. In 1927, Dudgeon and Patrick was the first one to used FNAC in diagnosing tuberculous lymphadenitis [9,10]. Material obtained was sufficient which correlated with the study by Rana et al. and Hemalatha et al. [9-11]. In this study, the majority of patients were between 15 and 30 years which correlated with the study by Gupta et al. and Badge et al. [12,13]. Tuberculous lymphadenopathy was the most common lesion in this study with female predominance in the second decade correlated with a study by Badge et al. [13]. The female predominance in this study was also comparable with studies conducted in this region where the tuberculous infection was common and other granulomatous lesions were less prevalent and the presence of granuloma on FNAC was highly suggestive of tuberculosis.

In a large-scale study on 1785 patients from Pakistan tuberculous lymphadenitis was the most common pathology followed by lymphoma on FNAC [14]. A similar study done in Peshawar by Khan et al. reported tuberculous lymphadenitis 37.2% in cervical lymph nodes [15]. This study's results regarding the prevalence of tuberculous lymphadenitis in cervical lymph nodes were also consistent with the study of Khan et al. [16]. Nahid and Hopewell from Turkey showed in their study that tuberculous lymphadenitis was the most common site for extrapulmonary TB [17]. In India, tuberculous lymphadenitis was the most common cause of lymphadenopathy [18]. However, data from developed countries showed that tuberculous lymphadenitis was found more in immigrants from endemic counties [19]. It was shown in different studies from France and Germany that tuberculous lymphadenitis had a prevalence of 70% to 75% in immigrants as compared to natives [20]. A study conducted in the USA showed that tuberculous lymphadenitis was more common in immigrants of Asia between 15 and 50 years [21-23].

Reactive hyperplasia (38.29%) in the present study was seen more in males (n=145) as compared to females (n=97). Detailed clinical examination, history, and other investigations were done to find the cause of hyperplasia. The findings of this study were consistent with the previous studies of Gupta et al. [17]. A study conducted in Malaysia showed reactive hyperplasia of lymph nodes was the most common pathology followed by metastasis [24]. In western countries, it was significantly lower, with some studies showing 1.6%. In a study of 452 cases from eastern KSA (Kingdom of Saudi Arabia), the nonspecific reactive disease was the most common pathology followed by granulomatous disease [25,26]. Our findings are consistent with Hemalatha et al. [11] which manifest metastatic cancer was found to be 3.63% in the present study and FNAC is adequate in diagnosing metastatic cancer as surgical biopsy. Squamous cell carcinoma, undifferentiated carcinoma, and adenocarcinoma were the most common cancers detected in FNAC of cervical lymph nodes. There were 13 cases of lymphoma, 10 cases had Hodgkin disease (6 males and 4 females, 1.58%) and 3 patients had non-Hodgkin disease including one male and two females (0.47%). All patients were between 15 and 40 years. Maximum patients (5) with lymphoma were between 21 and 30 years which shows consistency with previous studies.

## Conclusions

FNAC is the first line safe, quick, economical, and reliable investigation tool in diagnosing non-neoplastic and neoplastic lesions. Tuberculous cervical lymphadenitis was the most common pathology in our study. The modality of treatment can be focused on the results of FNAC in patients with lymphadenopathy. Our study highlighted various cytomorphological patterns of lymphadenopathy distribution with age and sex. Sensitivity and specificity of FNAC in superficial lymphadenopathy in the study were found to be 100%.
